# Adapting the WHO Safe Childbirth Checklist: A collaborative study in West Africa

**DOI:** 10.4102/jphia.v16i1.630

**Published:** 2025-01-31

**Authors:** Kadidiatou R. Kourouma, Tieba Millogo, Aissatou Diallo, Wambi M.E. Yaméogo, Marie L. Agbré-Yacé, Mamadou D. Baldé, Issaka Tiembré, Alexandre Delamou, Séni Kouanda

**Affiliations:** 1 National Public Health Institute of Côte d’Ivoire, Abidjan, Côte d’Ivoire; 2 Centre for Reproductive Health Research of Côte d’Ivoire, Abidjan, Côte d’Ivoire; 3 Department of Biomedical and Public Health, Institute for Research in Health Sciences (IRSS), Ouagadougou, Burkina Faso; 4 African Institute of Public Health (IASP), Ouagadougou, Burkina Faso; 5 Centre for Reproductive Health Research of Guinea, Conakry, Guinea; 6 Department of Biomedical and Public Health, Faculty of Medicine, University Félix Houphouët-Boigny, Abidjan, Côte d’Ivoire; 7 Department of Public Health, African Center of Excellence for the Prevention and Control of Communicable Diseases, Gamal Abdel Nasser University, Conakry, Guinea

**Keywords:** adaptation, Safe Childbirth Checklist, maternal health, neonatal health, implementation, West Africa

## Abstract

**Background:**

The World Health Organization Safe Childbirth Checklist (WHO SCC) was developed to increase the uptake of essential birth practices; however, only a few studies have adopted this process in French West African countries.

**Aim:**

This study aimed to describe the WHO SCC adaptation process across Burkina Faso, Côte d’Ivoire, and Guinea, and the lessons learned.

**Setting:**

Adaptation processes were conducted in the above-mentioned countries.

**Methods:**

From May 2022 to November 2022, a cross-country adaptation of the WHO SCC was carried out using a co-creation approach following a modified Delphi process. This process included the contextual adaptation of the tool by local technical advisory groups in each country based on national guidelines, the harmonisation and production of a single modified SCC by the Cross-Country Technical Advisory Group, a pre-test of the modified SCC, and adoption.

**Results:**

Minor modifications were made on 27 items. No items were deleted. Two items related to hand hygiene and the use of protective equipment were added at the ‘just before pushing’ pause point. The modified SCC implemented in each country consisted of 31 items, with variations observed in the timing of the monitoring signs when plotting the partograph. The tool was introduced following the A3 and kakemono formats.

**Conclusion:**

The study emphasised the importance of engaging all stakeholders and end users in the adaptation process for a sustainable use of the tool.

**Contribution:**

This collaborative effort between countries to develop a unified SCC highlights the importance of adaptation based on national guidelines and local contexts.

## Background

To mitigate the primary causes of maternal and neonatal mortality and morbidity, the World Health Organization (WHO) recommends the use of a safe childbirth checklist during delivery and the immediate postpartum period.^1^ The WHO Safe Childbirth Checklist (WHO SCC) is an effective, simple and easy-to-use tool, especially developed for low- and middle-income countries (LMICs), where resources for implementing extensive interventions may be limited. Evidence shows that healthcare providers enhance their delivery of cost-effective, evidence-based interventions during and immediately after childbirth using the WHO SCC.^2,3,4^ The 2015 original version of the WHO SCC comprises 29 items organised into four key pause points during childbirth: admission, just before pushing (or before caesarean section), soon after birth and before discharge. Each evidence-based item in the WHO SCC is a critical action that, if missed, can lead to severe harm.^5^ Therefore, the WHO SCC serves as a bedside tool for healthcare workers to improve the quality and safety of childbirth care by adopting proven, cost-effective and easy-to-perform interventions.^6^

Several sub-Saharan African countries were involved in the development and testing phases of the tool before its release for implementation by frontline healthcare workers.^7^ The WHO SCC remains generic, inviting stakeholders to adapt it to their local contexts to reflect specific priorities and health system characteristics.

Studies conducted in different countries in Africa (Rwanda and Namibia), Asia (Sri Lanka) and Latin America (Brazil),^2,7,8,9,10^ where the tool has been tested in delivery rooms, have emphasised the importance of context-specific adaptations whenever introducing the WHO SCC.^7^ If the context and WHO SCC interactions with other interventions are not considered, the effectiveness of the interventions carried out using the WHO SCC is likely to be compromised.^11^ Indeed, the adaptation of the WHO SCC and the involvement of all stakeholders in this process have emerged as the main recommendations of the WHO and several other studies.^1,7,11,13,14^

In Burkina Faso, Côte d’Ivoire and Guinea, a two-phase multicentre study is underway to assess the feasibility, acceptability and effectiveness of the WHO SCC.^15,16,17^ In the first phase, which examined the feasibility of WHO SCC adoption and its acceptability in Burkina Faso and Côte d’Ivoire,^16^ frontline healthcare workers were generally willing to use the WHO SCC during childbirth. However, they suggested that effective implementation would require the adaptation of the tool to the local context.^16^

During Phase 2 of the checklist project in Burkina Faso, Côte d’Ivoire and Guinea, a process adaptation of the WHO SCC was carried out with the stakeholders to develop a single modified version for implementation in the three countries. In the intervention phase, the modified WHO SCC will be introduced in regional hospitals using a matched-pair cluster randomised controlled trial design.^17^

To the best of our knowledge, this is the first adaptation of the WHO SCC in West Africa and the first cross-country collaboration to develop a single modified WHO SCC (mSCC). This study reported the process undertaken to adapt and validate the content of the mSCC for Burkina Faso, Côte d’Ivoire and Guinea, as well as the approach used to determine the appropriate formats of the tool to be introduced in regional hospitals according to their specific contexts.

## Research methods and design

### Study settings

This study focussed on three French West African countries that bear the heavy burden of poor childbirth outcomes: Burkina Faso, Guinea and Côte d’Ivoire. Burkina Faso and Guinea are classified as low-income countries by the World Bank, while Côte d’Ivoire is classified as an LMIC. All three countries have made significant progress in shifting deliveries from home to health facilities in recent years. However, the increased utilisation of healthcare facilities for deliveries has not led to the anticipated improvements in delivery outcomes. The maternal mortality ratio (MMR) has remained at an alarmingly high level, significantly deviating from global targets. The recent MMR estimates in Burkina Faso, Guinea and Côte d’Ivoire were 264, 550, and 385 per 100 000 live births, respectively. Similarly, the neonatal mortality rates remained elevated across these countries, with 18, 32, and 30 deaths per 1000 people in Burkina Faso, Guinea and Côte d’Ivoire, respectively.^18,19,20^

### Generic World Health Organization safe childbirth checklist

The generic WHO SCC, 2015 version, is composed of 29 items organised into four key pause points during facility-based childbirth^1^: upon admission, just before pushing (or before caesarean section), soon after birth and before discharge. The first pause point, on admission, is composed of eight items aimed at detecting and managing pre-existing complications, determining the need for referral to a higher level of care and preparing the mother (and her companion) for labour and delivery through improved interpersonal communication and health education. The second key pause point, just before pushing (or before caesarean section), include five items focussed on preparing the materials and drugs needed for the delivery. The third key pause point, soon after birth, is composed of nine items aimed at promptly identifying and managing immediate postpartum complications. It is also aimed at educating the mother (and her companion) about danger signs that necessitate seeking immediate help. The final pause point, at discharge, comprises seven items aimed at ensuring the health of the mother and newborn before discharge and adequately planning post-natal visits. During this phase, family planning options must be discussed and offered to the mother (and her companion). Additionally, educating them on the danger signs in both the mother and baby is essential for ensuring immediate skilled care. Each item is accompanied by a set of instructions drawn from the global guidelines on childbirth care.

### Co-creation and participatory design

To ensure a comprehensive adaptation of the checklist that incorporates input from various stakeholders involved in its use, the adaptation process is guided by a co-creation approach.^21,22,23^ Co-creation has the potential to increase the effectiveness of implementation and interventions.^24^

The co-creation process followed a participatory approach that combined top-down (expert opinion) and bottom-up (provider) approaches.^25^ In practice, this co-creation process for adapting the WHO SCC involved two entities: Local technical advisory groups (L-TAGs) in each country and the Cross-Country Technical Advisory Group (CC-TAG).

The L-TAGs comprised of experts, including a research team, selected in collaboration with the Ministry of Health in each country. The L-TAGs included 11, 19, and 13 experts from Burkina Faso, Côte d’Ivoire and Guinea, respectively. These experts were drawn from another division of the Ministry of Health specialising in maternal and child health, the Scientific Society of Gynaecology and Obstetrics, the Scientific Society of Paediatrics, and the Nurses Association and Implementation Partners. Most experts were medical doctors and midwives.

Meanwhile, the CC-TAG comprised of all the members of the research team across the three countries and one focal point of the L-TAG who was designated by others L-TAGs members.

This co-creation-based approach utilised a modified Delphi method, a structured group communication process that allows a group of individuals to address complex problems. This technique was used to gain consensus or agreement among specialists regarding the proposed problem rather than relying on a single professional to make a decision.^26^

### Description of the adaptation process phases

**FIGURE 1:**
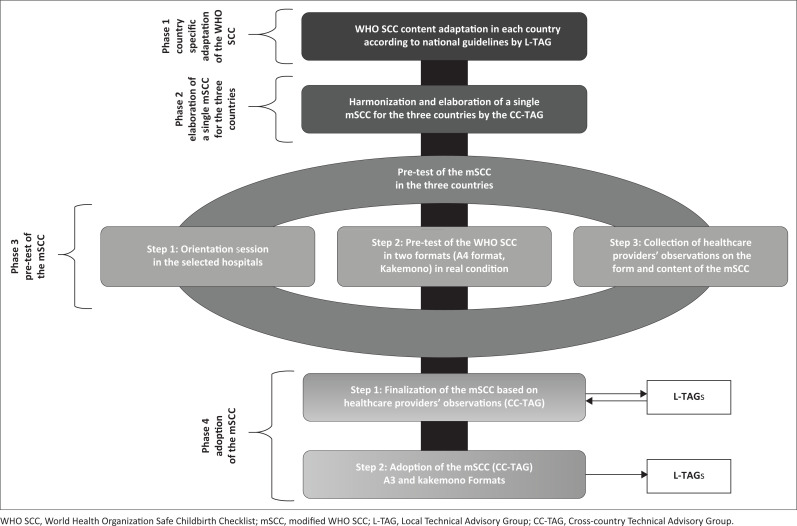
WHO Safe Childbirth Checklist adaptation process phases in Burkina Faso Cote d’Ivoire and Guinea.

The adaptation process took place from May to November 2022 and encompassed four (04) phases: (1) country-specific adaptation of the WHO SCC based on national guidelines by L-TAGs, (2) harmonisation and production of a single mSCC by the CC-TAG, (3) pre-test of the mSCC in the three countries and (4) adoption of the mSCC (Figure 1).

#### Phase 1: Country-specific adaptation of the World Health Organization Safe Childbirth Checklist

In each country, the L-TAG met in a 1-day workshop to revise and input the generic tool according to the national guidelines, starting with an introduction to the WHO SCC by the research team, followed by an individual assessment of the tool and plenary discussions.

Local technical advisory group members evaluated the WHO SCC based on relevance, clarity and sufficiency using a Likert scale from 1 to 4. Clarity referred to the ease of understanding the items and instructions, relevance addressed the items importance for checklist application and sufficiency related to how well the pause points covered EBPs.^27^

To reduce bias, a modified Delphi method was used, allowing experts to score independently without knowledge of others’ scores. After individual assessments, a plenary session facilitated consensus-building. Average scores for each item were discussed, focussing on those with scores below 4. Discussions aimed for equitable participation, leading to informed modifications based on consensus.

#### Phase 2: Elaboration of a single modified World Health Organization Safe Childbirth Checklist for the three countries

In July 2022, we conducted two virtual design workshops with the CC-TAG to achieve the following objectives:

- Harmonise and elaborate a single mSCC.

One week before the first meeting, all CC-TAG members received a synthesis of observations and country-specific modifications, which were then discussed based on the national guidelines and WHO directives to reach a consensus:

- Discuss the mSCC formats for introduction during the implementation phase.

During the meeting, CC-TAG members discussed the mSCC format for selected regional hospitals in the randomised trial and agreed to implement both A4 and kakemono formats.

#### Phase 3: Pre-test of the modified World Health Organization Safe Childbirth Checklist in each country

The mSCC was pre-tested in two health facilities per country over two days. Prior to the pre-test, the medical staff were oriented on the mSCC’s use and its importance in maternity care. Two formats were tested: A4 and kakemono formats. Healthcare providers pre-tested the tool on a maximum sample of five childbirth events per health facility and completed an open-ended questionnaire for feedback. After the pre-test, questionnaires were collected for analysis of observations and suggestions from healthcare providers.

#### Phase 4: Adoption of the modified World Health Organization Safe Childbirth Checklist

In the final phase, the comments and suggestions gathered during the pre-test were reviewed during a 2-day online restitution workshop conducted by the CC-TAG. Based on feedback from healthcare providers, adjustments were made to finalise the mSCC and maintain the formats proposed. The final version of the mSCC was approved by all members involved.

### Ethical considerations

Ethical approval to conduct this study was obtained from the National Ethics Review Committees of Burkina Faso (No. N°2020–12-273), Côte d’Ivoire (No. 165-20/MSHP/CNESVS-kp), and Guinea (No. N°104/CNERS/20), and the Research Ethics Review Committee of the WHO (No. ERC.0003400).

## Results

### Content adaptation of the modified World Health Organization Safe Childbirth Checklist

**FIGURE 2:**
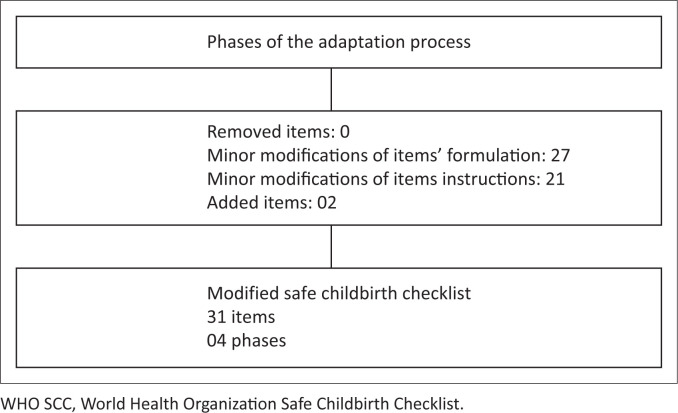
Flowchart of the WHO SCC adaptation content results.

Considering the different phases of content adaptation aligned with the national guidelines, the CC-TAG finally decided not to remove any items, add two new items and make minor changes to 27 items and 21 sets of instructions (Figure 2).

The decisions made during the content adaptation process are presented in Online Appendix 1. In terms of item relevance, based on the scores provided by the L-TAGs and discussions within the CC-TAGs, all items were deemed relevant.

To ensure clarity, minor modifications and clarifications were made to the items and instructions. All point pauses were related to these modifications and clarifications. These adjustments focussed on the referral process, partograph usage, conditions for administering antibiotic treatment to the parturient or mother, the criteria for determining abnormal bleeding, essential supplies for parturients and newborns, and identification of danger signs.

These modifications and clarifications were standardised across all countries, except for item 2, which was related to the partograph. In Côte d’Ivoire, in accordance with the national guidelines, the number and intensity of contractions, parturient heart rhythm, and foetal heart rhythm were recorded every 1 h. The parturient’s blood pressure was measured every 2 h, while the patient’s temperature was measured every 4 h. In Burkina Faso and Guinea, the national guidelines were aligned with those on the checklist, which included the monitoring of the number and intensity of contractions, parturient heart rhythm, and foetal heart rhythm every 30 min; blood pressure every 4 h; and temperature every 2 h.

Additional modifications focussed on rewording and replacing the term ‘mothers’ with ‘parturients’ at the first two pause points. The term ‘baby’ was also replaced with newborn at all pause points.

In terms of sufficiency, two items were added in the second pause point, ‘just before pushing’, These additional items, which were the same for all three countries, focussed on hand hygiene and the use of protective equipment for healthcare providers and assistants. Additionally, the title of the first pause point was changed to ‘on admission after initial examination’.

Participants in each country found the mSCC to be very useful during the pre-test, as it helped them have better control of their practices. They provided suggestions, which were incorporated to finalise the mSCC. The final versions of the mSCC used in Burkina Faso, Côte d’Ivoire and Guinea are presented in Online Appendix 2.

### Formats of the modified World Health Organization Safe Childbirth Checklist to be introduced during the implementation phase

#### A4 format

In the three countries, the participants agreed that the font size of the A4 format should be increased. Overall, they were satisfied with the colours used. In Guinea, the participants suggested that important elements should be highlighted in red or bold to attract the attention of healthcare providers and that background colours should not be used.

During the adoption phase, the CC-TAG decided that instead of the A4 format, an A3 format should be used in the regional hospitals, with the first two pause points on the front and the last two on the back. The A3 format is placed in the patient’s file or attached to a partograph.

#### Large display format

In general, participants in all three countries were satisfied with the colour, font size, design and format used. However, the layout of the large display format was a concern for both healthcare providers and the CC-TAG, as the size of the maternity ward varied from one health facility to another.

The CC-TAG decided that, in each country, the research team, in collaboration with the medical staff, found the most appropriate large display format and location for optimal use. In Côte d’Ivoire, where wall displays are forbidden, the large display format used was the kakemono format. In Guinea and Burkina Faso, the kakemono was introduced as a room divider.

## Discussion

This study describes the first cross-country adaptation of the WHO SCC in French West Africa through the establishment of national and cross-country TAGs. Indeed, any process of adapting, modifying and adopting the WHO SCC must be performed in collaboration with the research team, experts and all healthcare providers who may use the tool. All stakeholders must engage early in this process, not only to help make appropriate modifications but also to create a sense of ownership that is crucial for facilitating adoption and use in routine practice.^1,12^ This adaptation process with all stakeholders facilitates the initial implementation and successful uptake.^12^ A previous study that assessed the adaptation and validation of the WHO SCC worldwide showed that most participants reported adapting the WHO SCC to match local guidelines and protocols in the engagement phase (79.3%) and met with stakeholders to obtain a buy-in for the WHO SCC (75.9%).^28^ In addition, the tool must be tested prior to any mSCC rollout. Real-time feedback from healthcare providers is crucial for the successful adaptation of a checklist and its integration into the care process.

### Content adaptation

In our study, after completing all phases, the adaptation process of the mSCC led to the development of a unified checklist comprising 31 items to be used in Burkina Faso, Côte d’Ivoire and Guinea. Some differences were observed in the instructions regarding the times for monitoring signs when plotting partographs (item 2) during admission. For Burkina Faso, the times have remained the same as those of the WHO SCC. Instructions related to partographs were also modified in some countries, such as Brazil and Colombia, to suit their local contexts.^10,27^ In Colombia, for instance, blood pressure was measured every 2 h instead of every 4 h because the longer interval was deemed too infrequent to assess the impact of potential interventions. Conversely, temperature was measured every 4 h instead of every 2 h as fever during labour is considered rare; this parameter does not vary frequently enough to warrant more frequent checks.^27^

Other examples of adaptations to SCC content worldwide include the addition of clinical items (gestational age dating, the management of preterm labour and birth, respectful care practices, the appropriate use of corticosteroids and tocolytics, newborn anthropometrics, and kangaroo mother care) and operational items (confirmation practices, the names and signatures of people involved in care, supply inventory, discharge summary, and transportation arrangement).^28^

In some cases, the WHO SCC has also been translated into local languages to improve accessibility.^9,10,27,28^ In our process, this was not necessary as the official language in the three countries is French. A French version of the WHO SCC is available.

In addition to contextual adaptation, a crucial element for the healthcare providers’ acceptance of the mSCC is its format for integration into the healthcare system. The checklist should be user-friendly, well-designed, easily accessible and placed in visible locations.^7^ In our intervention study, the CC-TAG decided that the two formats to be introduced in the regional hospitals would be the A3 format attached to the partograph or the mother’s chart, and the large display format as a kakemono or a wall poster. Lessons learned from a global collaboration revealed that, according to end users and the implementation team, besides strong leadership, the characteristics of the tool and how the mSCC was implemented contributed positively to its use.^7^ In most studies conducted in other settings, the mSCC is formatted on a single page and integrated into existing tools, such as the mother’s chart,^5,7,8,28,29,30^ or displayed in the maternity ward.^7,9,28^ However, there are examples of the WHO Safe Surgery Checklist being incorporated into a mobile app and different versions created for physicians, midwives, and nurses.^28^ Regardless of the format, the introduction must be agreed upon with the end users for optimal routine use.

### Lessons learned and implications for the next steps

Stakeholders’ engagement is crucial to the success of the adaptation process. Additionally, not all adaptations to local contexts and personal preferences add value. If changes to the WHO SCC are likely to impact understanding and effectiveness, a team of experts must be able to arbitrate. The WHO SCC includes essential best practices that should not be removed. The mSCC must remain simple. If there are too many items, healthcare providers may consider this as an additional workload.

Buy-in is central to the successful implementation of the mSCC; therefore, healthcare providers must take ownership of the tool. To facilitate this, the research team must collaborate with the medical staff to adequately train them on the tool and its use, explain to the coach what is expected from them, describe the intervention and expected outcomes, and carry out formative supervision at the intervention sites during the implementation phase.

### Strengths and limitations of the adaptation process

The strength of this process lies in the engagement of stakeholders from the central level with end users; meanwhile some phases could not be carried out concomitantly as planned, particularly the adaptation by the L-TAGs and pre-tests. This lengthened the duration of the adaptation process and affected the start of other activities, including the implementation of the intervention initially planned for September 2022. In addition, while the study provides valuable insights for the three countries involved, the findings may have limited generalisability to other regions with different health system characteristics. However, countries wishing to carry out this adaptation process will be able to draw on the lessons learned from this collaboration.

## Conclusion

This cross-country adaptation process highlights the importance of engaging all stakeholders. The mSCCs obtained from a consensus in the three countries are expected to be used by healthcare providers in routine practice; however, modifying the WHO SCC to reflect the local context and national guidelines is insufficient. Strategies such as the mSCC launch, continuous engagement, and support through coaching and training should be considered when implementing mSCC.
